# Prognostic Value of Standard Base Excess in Sepsis‐Associated Acute Kidney Injury: A Retrospective Cohort Study

**DOI:** 10.1155/ijne/8851195

**Published:** 2026-09-22

**Authors:** Mingming Li, Yuxia Li, Jingru Lan, Mei Feng, Jun Lyu, Xuling Li

**Affiliations:** ^1^ Department of Intensive Care Unit, The First Affiliated Hospital of Jinan University, Guangzhou, China, jd120.com; ^2^ Clinical Research Center, The First Affiliated Hospital of Jinan University, Guangzhou, China, jd120.com; ^3^ Key Laboratory of Regenerative Medicine of Ministry of Education, Guangzhou, China; ^4^ Department of Urology, The First Affiliated Hospital of Jinan University, Guangzhou, China, jd120.com

**Keywords:** MIMIC-IV database, mortality, sepsis-associated acute kidney injury, standard base excess

## Abstract

**Background:**

Standard base excess (SBE) is a pivotal indicator of metabolic acid–base status and has been associated with adverse outcomes in critically ill patients. Nevertheless, the evidence regarding its association with mortality risk in sepsis‐associated acute kidney injury (SA‐AKI) remains limited.

**Methods:**

The present study utilized the MIMIC‐IV database and comprised 16,842 adult patients with SA‐AKI. Patients were stratified into quartiles based on SBE levels. The association between SBE and 28‐day and 180‐day all‐cause mortality was evaluated using Cox proportional hazards models. Restricted cubic spline analysis was performed to assess potential nonlinear relationships. Multivariable adjustments and subgroup analyses were conducted to ensure the robustness of the findings.

**Results:**

Multivariate Cox regression analysis demonstrated that patients in the higher SBE quartiles had significantly lower risks of 28‐day and 180‐day mortality compared to those in the lowest quartile. Kaplan–Meier survival curves and restricted cubic spline analysis further confirmed a significant association between SBE levels and mortality risk, with evidence of a nonlinear relationship. Subgroup analyses showed different interaction effects between the primary and secondary outcomes.

**Conclusions:**

SBE was associated with mortality risk in patients with SA‐AKI, with lower levels of SBE being associated with worse outcomes. As a routine blood gas parameter, SBE may serve as a useful indicator for risk stratification and prognostic assessment in this population.

## 1. Introduction

Sepsis‐associated acute kidney injury (SA‐AKI) is among the most prevalent and challenging complications in critical care [1]. As delineated by the 28th Acute Disease Quality Initiative (ADQI), SA‐AKI is defined as acute kidney injury (AKI) occurring in the context of sepsis, driven by a complex interplay of systemic inflammation, renal microcirculatory dysfunction, oxidative stress, and immune dysregulation [2]. Mortality rates in patients with SA‐AKI are significantly higher than in those without SA‐AKI, with in‐hospital mortality ranging from 11% to 77% [2, 3]. The long‐term prognosis is also poor, with a significant proportion of patients at risk of progressing to chronic kidney disease (CKD) or end‐stage renal disease (ESKD). This has a considerable impact on the quality of life and places a substantial burden on healthcare systems and society [4, 5].

In this context, the effective assessment of the prognostic risk in patients with SA‐AKI has become a critical focus in clinical practice. Base excess (BE), a frequently employed marker of metabolic acid–base disturbances, is widely utilized to reflect the severity of nonrespiratory acidosis or alkalosis [6]. However, the reliability and representativeness of BE measurements are potentially limited by multiple physiological variables, including concentrations of plasma proteins and hemoglobin. Conversely, the standard base excess (SBE), as determined by the Van Slyke formula, is advocated as the optimal clinical metric, exhibiting superior in vivo precision and reliability [7]. A number of earlier studies have demonstrated that BE, as an integrated indicator of acid–base balance, is closely associated with mortality, organ dysfunction, and clinical outcomes across a range of critical illnesses. These findings underscore its potential as a prognostic marker [8–10].

However, there is a notable absence of systematic research on the clinical relevance of SBE in the SA‐AKI population. It is evident that SA‐AKI is frequently accompanied by profound acid–base disturbances and multiorgan dysfunction. In such cases, SBE can serve as a straightforward metabolic marker, aiding in the evaluation of disease severity and mortality risk. The objective of this study is to examine the relationship between SBE levels and both short‐term and long‐term all‐cause mortality in patients with SA‐AKI. A large‐scale clinical database will be utilized to establish novel reference criteria for prognostic assessment in this high‐risk population.

## 2. Methods

### 2.1. Study Design and Data Source

The present retrospective cohort study, based on the Medical Information Mart for Intensive Care IV (MIMIC‐IV, version 3.1) database, aims to examine the association between SBE and both short‐term (28‐day) and long‐term (180‐day) all‐cause mortality in patients with SA‐AKI. The MIMIC‐IV database, developed collaboratively by the Massachusetts Institute of Technology (MIT) and Beth Israel Deaconess Medical Center, contains comprehensive clinical data on over 90,000 adult intensive care unit (ICU) admissions between 2008 and 2022, including demographics, vital signs, laboratory results, medication records, diagnostic codes, and mortality outcomes. MIMIC‐IV is a widely used open‐access clinical big data resource, which offers a robust foundation for observational research [11, 12]. The present study is conducted in strict accordance with the Strengthening the Reporting of Observational Studies in Epidemiology (STROBE) guidelines [13] and adheres to the ethical standards set forth in the Declaration of Helsinki. As all patient data in the MIMIC‐IV database are deidentified and appropriate data usage permissions have been obtained, additional ethical approval was not required.

### 2.2. Study Population

The present study comprised patients admitted to the ICU between 2008 and 2022 from the MIMIC‐IV database. The inclusion criteria comprised the following: (1) was over 18 years of age; (2) had been diagnosed with SA‐AKI according to the consensus criteria of the 28th ADQI [2]; and (3) only the first ICU admission was considered to prevent duplicate counting. SA‐AKI was defined as AKI occurring in the context of confirmed sepsis, with sepsis identified per Sepsis‐3 criteria as infection accompanied by organ dysfunction (Sequential Organ Failure Assessment [SOFA] score increase ≥ 2) [14] and AKI defined according to Kidney Disease: Improving Global Outcomes (KDIGO) guidelines [15].

In order to enhance the quality of the data and the analytical reliability, patients were excluded for the following reasons: Firstly, patients who had an ICU stay shorter than 24 h were excluded, thus minimizing the impact of brief admissions on outcomes. Secondly, patients who lacked key laboratory data, including arterial blood bicarbonate and pH values, necessary for calculating SBE, were also excluded (Supporting Figure 1).

### 2.3. Data Extraction

The present study extracted the necessary clinical data from the MIMIC‐IV (version 3.1) database using Structured Query Language (SQL). The extracted variables encompassed demographic characteristics, illness severity scores, vital signs, laboratory results, comorbidities, and clinical outcomes. For variables that exhibited multiple measurements (e.g., vital signs and laboratory tests), the initial value recorded after ICU admission was utilized to minimize bias and standardize the assessment timeframe [16].

### 2.4. SBE

SBE is a widely utilized arterial blood gas parameter that is employed for the assessment of metabolic acid–base status. This is achieved by quantifying the excess or deficit of buffering bases in the blood, thereby indicating the presence and severity of metabolic acidosis or alkalosis [17]. The SBE was calculated using the following formula: SBE (mmol/L) = HCO_3_^-^ (mmol/L) – 24.8 + 16.2 × (pH‐7.4) [7]. The pH and HCO_3_^-^ values were extracted from arterial blood gas measurements in the MIMIC‐IV database, and SBE was subsequently calculated. The earliest available measurements within 24 h after ICU admission were used to represent the initial SBE level. Patients were stratified into quartiles (Q1–Q4), and the associations between SBE levels and short‐term and long‐term all‐cause mortality were assessed.

### 2.5. Outcomes and Follow‐Up

The primary outcome of this study was a 28‐day all‐cause mortality, with a 180‐day all‐cause mortality serving as the secondary outcome. Mortality data were obtained from the date of death (DOD) recorded in the MIMIC‐IV database. The temporal parameters of follow‐up time and survival status were calculated from the initial ICU admission time (icu_intime). The definition of survival duration was as the interval between DOD and icu_intime in days. For patients without a recorded death, the follow‐up process was concluded at the last documented discharge date or the final postdischarge follow‐up available in the database.

### 2.6. Statistical Analysis

Statistical analyses were conducted using R (version 4.2.2), with significance defined as *p* < 0.05.

The baseline characteristics were summarized in a descriptive manner. Continuous variables were presented as the mean ± standard deviation (SD) or as the median with interquartile range (M [Q_1_, Q_3_]) based on the distribution, while categorical variables were reported as frequencies and percentages. Between‐group comparisons employed one‐way ANOVA for normally distributed continuous variables, the Kruskal–Wallis H test for non‐normal continuous variables, and the chi‐squared test (*χ* ^2^) for categorical variables. Survival analyses were conducted utilizing multivariate Cox proportional hazards models to evaluate the association between SBE levels and 28‐day and 180‐day all‐cause mortality. Models were adjusted for prognostically relevant confounders, including demographics, comorbidities, illness severity scores, and laboratory parameters (Supporting Table 1). SBE was analyzed both as a categorical variable by quartiles and as a continuous variable using restricted cubic spline (RCS) regression to assess potential nonlinear relationships with mortality risk.

Furthermore, Kaplan–Meier (KM) survival curves were generated for each SBE quartile group, with log‐rank tests applied to compare survival differences across groups, thereby visually elucidating the relationship between SBE levels and patient outcomes. All findings were expressed as hazard ratios (HRs) with corresponding 95% confidence intervals (95% CIs).

### 2.7. Subgroup Analyses

In order to further evaluate the stability and consistency of the association between SBE and all‐cause mortality across diverse populations, exploratory subgroup analyses were performed. The predefined subgroups encompassed four clinical variables: age (≤ 67 vs. > 67 years), gender (male vs. female), race (white vs. nonwhite), and SOFA score (≤ 10 vs. > 10). The age cutoffs were determined based on the median age of the cohort, while the SOFA threshold of 10 was identified as the optimal discriminator using the “survminer” package in R.

Subsequently, separate multivariate Cox proportional hazards models were constructed for each subgroup in order to assess the relationship between SBE quartiles and 28‐day and 180‐day mortality. HRs with 95% CIs were reported for each subgroup. Interaction terms were incorporated into the study to ascertain statistically significant effect modifications, thereby identifying populations in which the impact of SBE on mortality may vary.

### 2.8. Missing Value

The missing data rate for variables included in the final analyses was below 10% (Supporting Figure 2). Variables with substantial missingness, including neutrophils (16.38%), monocytes (16.65%), lymphocytes (16.95%), lactate (30.91%), and height (34.47%), were excluded. In order to mitigate the effects of missing data while maintaining the original sample size, multiple imputation was employed. Specifically, the random forest method within the R “mice” package was utilized to impute missing covariate values, with imputation restricted to predictor variables; outcome variables were not imputed. All statistical analyses were performed on the fully imputed dataset in order to ensure the validity and robustness of the results.

## 3. Results

### 3.1. Baseline Information of Patients With SA‐AKI

The present study comprised 16,842 patients who were admitted to the ICU for the first time and met the diagnostic criteria for SA‐AKI. Patients were stratified into quartiles based on SBE levels. Table 1 provides a synopsis of the demographic characteristics, disease severity scores, physiological parameters, and treatment‐related factors across the SBE groups. The mean age of the cohort was 67.55 ± 15.27 years, with females accounting for 41.15% of the population. A significant upward trend in age was observed with increasing SBE quartiles (*H* = 79.847, *p* < 0.001). Statistically significant differences were also noted in gender, race, and year of admission across quartiles. A notable variation was observed in the comorbidity burden and illness severity scores according to the severity of SBE levels. With regard to therapeutic interventions, patients in the lower SBE quartiles exhibited significantly higher rates of mechanical ventilation, renal replacement therapy (RRT), vasopressor use, septic shock, and CKD (all *p* < 0.001).

**TABLE 1:** Descriptive statistics of the study population.

Variables	Total (*n* = 16,842)	Quartile 1 (*n* = 4183)	Quartile 2 (*n* = 4163)	Quartile 3 (*n* = 4219)	Quartile 4 (*n* = 4277)	Statistics	*p* value
Age, mean ± SD	67.55 ± 15.27	65.90 ± 16.60	67.46 ± 15.63	67.41 ± 14.50	69.41 ± 14.06	*H* = 79.847	< 0.001
Sex, *n*(%)						χ^2^ = 96.506	< 0.001
Male	9912 (58.85)	2248 (53.74)	2495 (59.93)	2702 (64.04)	2467 (57.68)		
Female	6930 (41.15)	1935 (46.26)	1668 (40.07)	1517 (35.96)	1810 (42.32)		
Race, *n*(%)						χ^2^ = 172.375	< 0.001
Asian	440 (2.61)	127 (3.04)	118 (2.83)	105 (2.49)	90 (2.1)		
Black	1699 (10.09)	486 (11.62)	400 (9.61)	350 (8.3)	463 (10.83)		
Hispanic/Latino	565 (3.35)	163 (3.9)	127 (3.05)	139 (3.29)	136 (3.18)		
Other	2901 (17.22)	922 (22.04)	706 (16.96)	672 (15.93)	601 (14.05)		
White	11,237 (66.72)	2485 (59.41)	2812 (67.55)	2953 (69.99)	2987 (69.84)		
Admission year, *n*(%)						χ^2^ = 646.595	< 0.001
2008–2010	5624 (33.39)	1089 (26.03)	1300 (31.23)	1400 (33.18)	1835 (42.9)		
2011–2013	3639 (21.61)	790 (18.89)	860 (20.66)	990 (23.47)	999 (23.36)		
2014–2016	3523 (20.92)	821 (19.63)	959 (23.04)	972 (23.04)	771 (18.03)		
2017–2019	2502 (14.86)	865 (20.68)	656 (15.76)	544 (12.89)	437 (10.22)		
2020–2022	1554 (9.23)	618 (14.77)	388 (9.32)	313 (7.42)	235 (5.49)		
ICU type, *n*(%)						χ^2^ = 950.090	< 0.001
CCU	1704 (10.12)	502 (12)	408 (9.8)	363 (8.6)	431 (10.08)		
CVICU	3941 (23.4)	330 (7.89)	1183 (28.42)	1399 (33.16)	1029 (24.06)		
MICU	4379 (26)	1493 (35.69)	917 (22.03)	865 (20.5)	1104 (25.81)		
MICU/SICU	2755 (16.36)	781 (18.67)	617 (14.82)	592 (14.03)	765 (17.89)		
Other ICU	4063 (24.12)	1077 (25.75)	1038 (24.93)	1000 (23.7)	948 (22.17)		
Weight, kg, Mean ± SD	83.43 ± 22.60	81.16 ± 21.99	82.77 ± 21.60	84.67 ± 22.18	85.06 ± 24.29	*H* = 83.715	< 0.001
CCI, Mean ± SD	5.55 ± 2.97	5.83 ± 3.14	5.46 ± 3.03	5.31 ± 2.94	5.61 ± 2.74	*H* = 79.279	< 0.001
SOFA, Mean ± SD	6.55 ± 3.52	8.08 ± 3.95	6.40 ± 3.34	5.91 ± 3.20	5.84 ± 3.08	*H* = 945.893	< 0.001
APSIII, Mean ± SD	52.64 ± 22.61	63.62 ± 24.64	51.04 ± 21.70	47.34 ± 20.66	48.69 ± 19.39	*H* = 1323.889	< 0.001
Temperature, °C, Mean ± SD	36.87 ± 0.52	36.83 ± 0.57	36.87 ± 0.53	36.89 ± 0.50	36.88 ± 0.50	*H* = 27.498	< 0.001
Heart rate, bpm, Mean ± SD	86.95 ± 16.17	89.90 ± 17.75	86.56 ± 15.96	85.65 ± 15.08	85.73 ± 15.41	*H* = 185.444	< 0.001
Resp rate, bpm, Mean ± SD	19.71 ± 4.10	20.66 ± 4.55	19.52 ± 4.00	19.25 ± 3.86	19.42 ± 3.81	*H* = 266.225	< 0.001
MBP, mmHg, Mean ± SD	76.10 ± 9.48	75.98 ± 10.09	75.91 ± 9.11	76.39 ± 9.17	76.13 ± 9.51	*H* = 9.501	0.023
SpO_2_, Mean ± SD	97.11 ± 2.00	97.05 ± 2.11	97.26 ± 1.89	97.21 ± 1.90	96.91 ± 2.08	*H* = 64.387	< 0.001
Glucose, mg/dL, M(Q_1_, Q_3_)	129.00 (105.00–170.00)	142.00 (109.00–211.50)	129.00 (107.00–166.00)	125.00 (104.00–160.00)	123.00 (102.00–155.00)	*H* = 324.008	< 0.001
Sodium, mEq/L, Mean ± SD	137.95 ± 5.62	136.73 ± 6.72	137.95 ± 5.37	138.21 ± 4.80	138.88 ± 5.21	*H* = 353.974	< 0.001
Potassium, mEq/L, Mean ± SD	4.40 ± 0.90	4.68 ± 1.13	4.41 ± 0.85	4.29 ± 0.74	4.23 ± 0.75	*H* = 466.448	< 0.001
Septic shock, *n*(%)						χ^2^ = 643.434	< 0.001
No	13,466 (79.95)	2796 (66.84)	3366 (80.86)	3611 (85.59)	3693 (86.35)		
Yes	3376 (20.05)	1387 (33.16)	797 (19.14)	608 (14.41)	584 (13.65)		
CKD, *n*(%)						χ^2^ = 181.748	< 0.001
No	12,315 (73.12)	2739 (65.48)	3075 (73.87)	3280 (77.74)	3221 (75.31)		
Yes	4527 (26.88)	1444 (34.52)	1088 (26.13)	939 (22.26)	1056 (24.69)		
MV, *n*(%)						χ^2^ = 78.048	< 0.001
No	7802 (46.32)	2183 (52.19)	1851 (44.46)	1847 (43.78)	1921 (44.91)		
Yes	9040 (53.68)	2000 (47.81)	2312 (55.54)	2372 (56.22)	2356 (55.09)		
RRT, *n*(%)						χ^2^ = 234.909	< 0.001
No	16,025 (95.15)	3797 (90.77)	4005 (96.2)	4097 (97.11)	4126 (96.47)		
Yes	817 (4.85)	386 (9.23)	158 (3.8)	122 (2.89)	151 (3.53)		
Vasopressor, *n*(%)						χ^2^ = 68.782	< 0.001
No	8625 (51.21)	2041 (48.79)	2038 (48.96)	2126 (50.39)	2420 (56.58)		
Yes	8217 (48.79)	2142 (51.21)	2125 (51.04)	2093 (49.61)	1857 (43.42)		
28‐day mortality, *n*(%)						χ^2^ = 471.730	< 0.001
No	12,959 (76.94)	2717 (64.95)	3285 (78.91)	3505 (83.08)	3452 (80.71)		
Yes	3883 (23.06)	1466 (35.05)	878 (21.09)	714 (16.92)	825 (19.29)		
180‐day mortality, *n*(%)						χ^2^ = 391.866	< 0.001
No	11,291 (67.04)	2298 (54.94)	2932 (70.43)	3111 (73.74)	2950 (68.97)		
Yes	5551 (32.96)	1885 (45.06)	1231 (29.57)	1108 (26.26)	1327 (31.03)		

*Note:* Participants were categorized into quartiles based on serum SBE levels as follows: Quartile 1 (SBE ≤ −5.42; range: −29.7 to −5.42), Quartile 2 (−5.42 < SBE ≤ −1.96), Quartile 3 (−1.96 < SBE ≤ 1.04), Quartile 4 (SBE > 1.04; range: 1.04–25.7). M: Median; Q_1_: 1st Quartile; Q_3_: 3rd Quartile; χ^2^: Chi‐squared test; H: Kruskal–Wallis H test; CVICU: Cardiovascular Intensive Care Unit; SpO_2_: peripheral capillary oxygen saturation.

Abbreviations: APSIII, Acute Physiology Score III; CCI, Charlson Comorbidity Index; CCU, Coronary Care Unit; CKD, chronic kidney disease; ICU, intensive care unit; MBP, mean blood pressure; MICU, Medical Intensive Care Unit; MV, mechanical ventilation; RRT, renal replacement therapy; SD, standard deviation; SICU, Surgical Intensive Care Unit; SOFA, Sequential Organ Failure Assessment.

### 3.2. Primary and Secondary Outcomes

Patients in the Q1 group, representing the lowest SBE levels, exhibited a 28‐day mortality rate of 35.05%, which was significantly higher than that of the other groups. The lowest rate was observed in the Q3 group (16.92%). A similar pattern was noted for 180‐day mortality, with Q1 at 45.06% and Q3 at 26.26% (both *p* < 0.001).

The application of a Cox proportional hazards regression model revealed a statistically significant association between the higher quartiles and a reduced risk of both 28‐day and 180‐day all‐cause mortality, when compared with the lowest SBE quartile (Q1). These findings are outlined in Table 2. Following adjustment for confounding variables, including age, gender, race, Charlson Comorbidity Index (CCI), SOFA score, Acute Physiology Score III (APSIII) score, mechanical ventilation, RRT, vasopressor use, hypertension, diabetes, septic shock, and CKD, the adjusted HRs for 28‐day mortality in Q2, Q3, and Q4 were determined to be 0. The results of the study indicated that the prevalence of the condition increased with age, with an odds ratio of 81 (95% CI: 0.74–0.88), 0.73 (95% CI: 0.66–0.80), and 0.81 (95% CI: 0.74–0.88), respectively. A similar pattern was observed for 180‐day mortality. The findings indicate an independent inverse association between higher SBE levels and reduced short‐ and long‐term mortality risks, with all differences reaching statistical significance.

**TABLE 2:** Cox regression modeling results for the primary and secondary outcomes.

Variable	Unadjusted analysis		Adjusted analysis	
	HR (95% CI)	*p* value	aHR (95% CI)	*p* value
Primary outcome				
Quartile 1	Reference		Reference	
Quartile 2	0.53 (0.49, 0.58)	< 0.001^∗^	0.81 (0.74, 0.88)	< 0.001^∗^
Quartile 3	0.42 (0.38, 0.45)	< 0.001^∗^	0.73 (0.66, 0.80)	< 0.001^∗^
Quartile 4	0.48 (0.44, 0.52)	< 0.001^∗^	0.81 (0.74, 0.88)	< 0.001^∗^
Secondary outcome				
Quartile 1	Reference		Reference	
Quartile 2	0.56 (0.52, 0.60)	< 0.001^∗^	0.81 (0.75, 0.87)	< 0.001^∗^
Quartile 3	0.48 (0.45, 0.52)	< 0.001^∗^	0.80 (0.74, 0.86)	< 0.001^∗^
Quartile 4	0.58 (0.54, 0.62)	< 0.001^∗^	0.91 (0.85, 0.98)	0.018^∗^

*Note:* Participants were categorized into quartiles based on serum SBE levels as follows: Quartile 1 (SBE ≤ −5.42; range: −29.7 to −5.42), Quartile 2 (−5.42 < SBE ≤ −1.96), Quartile 3 (−1.96 < SBE ≤ 1.04), Quartile 4 (SBE > 1.04; range: 1.04–25.7). Cox proportional hazards regression models were used to calculate hazard ratios (HRs) with 95% confidence intervals (CIs). All models were adjusted for age, sex, race, Charlson Comorbidity Index (CCI), Sequential Organ Failure Assessment (SOFA), Acute Physiology Score III (APSIII), mechanical ventilation, renal replacement therapy, vasopressor use, hypertension, diabetes, septic shock, and chronic kidney disease.

Abbreviations: aHRs, adjusted hazard ratios; CIs, confidence intervals; HR, hazard ratio.

^∗^Significant difference between patients in four groups (*p* < 0.05).

**FIGURE 1:**
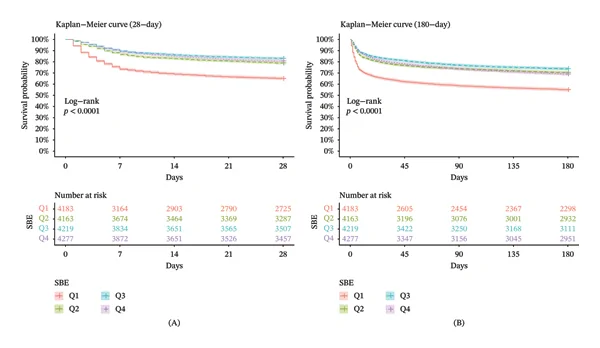
Kaplan–Meier survival curves between groups. Abbreviations: SBE: standard base excess; Q1: Quartile 1; Q2: Quartile 2; Q3: Quartile 3; Q4: Quartile 4. Note: (A) Kaplan–Meier survival curves (28 day). (B) Kaplan–Meier survival curves (180 day). The log‐rank test indicates a statistically significant difference in survival across the quartiles (*p* < 0.0001).

**FIGURE 2:**
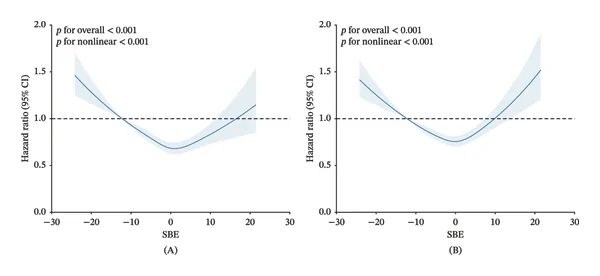
Restricted cubic spline analysis. Note: (A) Restricted cubic spline curves (28 day). (B) Restricted cubic spline curves (180 day). Models were adjusted for age, sex, race, Charlson Comorbidity Index (CCI), sequential organ failure assessment (SOFA), Acute Physiology Score III (APSIII), mechanical ventilation, renal replacement therapy, vasopressor use, hypertension, diabetes, septic shock, and chronic kidney disease.

KM survival curves further demonstrated significant differences in survival across SBE quartile groups, with patients in the Q1 group exhibiting markedly lower survival rates during both the 28‐day and 180‐day follow‐up periods (Figure 1, log‐rank test *p* < 0.0001). Furthermore, RCS analysis (Figure 2) revealed a U‐shaped nonlinear association between SBE and mortality risk, indicating that increases in SBE within a certain range may offer a protective effect by reducing the risk of death.

### 3.3. Subgroup Analyses

In subgroup analyses, the inverse correlation between SBE and mortality risk remained consistent across most strata (Table 3 and Supporting Table 2). Higher SBE levels were associated with a lower mortality risk. Interaction analyses identified significant interactions with age, sex, and SOFA score for both the primary and secondary outcomes.

**TABLE 3:** Subgroup analysis results in primary outcome.

Subgroups	SBE	HR (95% CI)	*p* value	p For interaction
*Age*				
< 67	Quartile 1	Reference		
	Quartile 2	0.80 (0.69–0.92)	0.002^∗^	0.492
	Quartile 3	0.69 (0.59–0.81)	< 0.001^∗^	0.233
	Quartile 4	0.71 (0.61–0.83)	< 0.001^∗^	0.012

≥ 67	Quartile 1	Reference		
	Quartile 2	0.84 (0.75–0.94)	0.002^∗^	0.492
	Quartile 3	0.77 (0.69–0.87)	< 0.001^∗^	0.233
	Quartile 4	0.89 (0.80–1.00)	0.043^∗^	0.012

*Sex*				
Female	Quartile 1	Reference		
	Quartile 2	0.80 (0.70–0.91)	0.001^∗^	0.670
	Quartile 3	0.77 (0.66–0.88)	< 0.001^∗^	0.109
	Quartile 4	0.81 (0.71–0.92)	0.002^∗^	0.380

Male	Quartile 1	Reference		
	Quartile 2	0.81 (0.73–0.91)	< 0.001^∗^	0.670
	Quartile 3	0.70 (0.62–0.80)	< 0.001^∗^	0.109
	Quartile 4	0.80 (0.71–0.91)	< 0.001^∗^	0.380

*Race*				
White	Quartile 1	Reference		
	Quartile 2	0.76 (0.68–0.84)	< 0.001^∗^	0.010
	Quartile 3	0.75 (0.66–0.84)	< 0.001^∗^	0.982
	Quartile 4	0.82 (0.74–0.92)	0.001^∗^	0.919

Others	Quartile 1	Reference		
	Quartile 2	0.88 (0.77–1.01)	0.640	0.010
	Quartile 3	0.68 (0.58–0.79)	< 0.001^∗^	0.982
	Quartile 4	0.76 (0.66–0.88)	< 0.001^∗^	0.919

*SOFA*				
< 10	Quartile 1	Reference		
	Quartile 2	0.86 (0.77–0.96)	0.008^∗^	0.186
	Quartile 3	0.77 (0.69–0.87)	< 0.001^∗^	0.241
	Quartile 4	0.86 (0.77–0.96)	0.007^∗^	0.220

≥ 10	Quartile 1	Reference		
	Quartile 2	0.75 (0.65–0.86)	< 0.001^∗^	0.186
	Quartile 3	0.66 (0.56–0.77)	< 0.001^∗^	0.241
	Quartile 4	0.73 (0.62–0.85)	< 0.001^∗^	0.220

*Note:* Participants were categorized into quartiles based on serum SBE levels as follows: Quartile 1 (SBE ≤ −5.42; range: −29.7 to −5.42), Quartile 2 (−5.42 < SBE ≤ −1.96), Quartile 3 (−1.96 < SBE ≤ 1.04), Quartile 4 (SBE > 1.04; range: 1.04–25.7).

Abbreviations: CIs, confidence intervals; HR, hazard ratio; SBE, standard base excess; SOFA, sequential organ failure assessment.

^∗^Significant difference between patients in four groups (*p* < 0.05).

Specifically, elevated SBE was associated with a more substantial reduction in mortality among patients younger than 67 years, whereas the protective effect in the Q4 group was diminished in those aged ≥ 67 years (*p* for interaction = 0.012 for 28‐day mortality; *p* = 0.011 for 180‐day mortality). In the analysis based on sex, no significant interaction was observed for 28‐day mortality (*p* for interaction = 0.380), whereas a borderline significant interaction was observed for 180‐day mortality (*p* for interaction = 0.049). Furthermore, patients with SOFA scores of 10 or more experienced a greater survival benefit from higher SBE levels, with the Q4 group showing a significant reduction in 180‐day mortality (*p* for interaction = 0.013), whereas no significant interaction was observed for 28‐day mortality (*p* for interaction = 0.220).

## 4. Discussion

The present study utilized a large‐scale critical care database to systematically evaluate the association between SBE at ICU admission and both short‐term and long‐term mortality risks in patients with SA‐AKI. The findings indicated a significant correlation between SBE levels and both 28‐day and 180‐day mortality, with the correlation persisting even after multivariable adjustment. Furthermore, the RBC model indicated a nonlinear relationship between SBE and mortality risk, suggesting that the association between SBE levels and mortality was not simply linear across the observed range. This finding highlights the potential prognostic relevance of SBE as a continuous marker of metabolic acid–base disturbances in patients with SA‐AKI.

SBE is a pivotal indicator for evaluating metabolic acid–base status, reflecting the balance of buffering alkali in the blood independent of respiratory influence [17]. A reduced SBE is indicative of metabolic acidosis, which is frequently associated with pathological states such as impaired tissue perfusion, lactic acid accumulation, renal dysfunction, and heightened inflammatory responses in critically ill patients [18, 19]. In patients with SA‐AKI, metabolic acidosis may result from multiple mechanisms, including lactic acidosis caused by tissue hypoperfusion and uremic acidosis due to impaired renal acid excretion and accumulation of unmeasured anions. As a high‐risk population, patients with SA‐AKI frequently exhibit profound metabolic derangements and internal environment disturbances [20]. Furthermore, alkalosis has been demonstrated to disrupt tissue oxygenation and impair ion channel function, thereby compromising cellular metabolism [21]. These findings emphasize the significance of maintaining SBE within an optimal range to preserve homeostasis and support organ function. The study also revealed that the prognostic value of SBE was more pronounced in specific subgroups, particularly among younger patients, those with elevated SOFA scores, and male patients. The possible explanations for this finding include greater metabolic resilience and heightened sensitivity to acid–base disturbances observed in younger individuals and the higher prevalence of metabolic acidosis in patients with severe multiorgan dysfunction, as reflected by elevated SOFA scores. Furthermore, biological sex is recognized as a risk factor for AKI, potentially influenced by hormonal differences between males and females [22]. The subgroup findings emphasize the clinical significance of enhanced dynamic monitoring of SBE in high‐risk populations to facilitate timely intervention and optimize outcomes.

Of particular significance is the fact that, in its capacity as a reversible and modifiable physiological marker, SBE holds a clinical value not only in the context of risk stratification but also in guiding therapeutic interventions. The extant evidence would appear to suggest that timely recognition and correction of metabolic acidosis can enhance organ perfusion, potentially mitigating the progression of AKI and preventing multiorgan dysfunction [23]. In cases of markedly reduced SBE, clinicians should evaluate and address potential causes of metabolic acidosis, such as impaired perfusion and lactate accumulation [19, 24, 25]. Nursing interventions are equally vital, encompassing increased frequency of blood gas monitoring, prompt evaluation of acid–base status, coordination with medical orders for individualized fluid and nutritional management, and continuous monitoring of renal function to facilitate early detection and intervention.

A review of the extant literature reveals a notable gap in research on SBE across diverse patient populations. The majority of extant studies have focused on blood gas parameters, including BE, lactate, and pH, as components of critical care scoring systems [8, 26, 27]. These systems are utilized to evaluate the relationship between acid–base balance and mortality risk. For instance, a correlation has been demonstrated between reductions in BE and an increased mortality rate in surgical critical care, sepsis, and postcardiac surgery settings [28–30]. However, research into the use of SBE as an independent prognostic indicator, particularly in populations with kidney injury, remains limited. This study extends the clinical utility of SBE by quantifying its predictive value for outcomes in SA‐AKI and confirming its nonlinear association with mortality, thereby contributing to the refinement of prognostic assessment in critically ill patients.

Notwithstanding the significant clinical ramifications of this study, it is imperative to acknowledge its several limitations. Firstly, it should be noted that this was a single‐center, retrospective observational study. Despite extensive adjustments being made for potential confounders, the possibility of residual confounding cannot be excluded, and unmeasured variables may still influence the observed association between SBE and patient outcomes. In particular, serum lactate was not included in the multivariable models due to its high missing rate (30.91%), which may have limited the assessment of the independent prognostic value of SBE. Additionally, excluding patients with ICU stays shorter than 24 h may have reduced the representation of early mortality cases with severe metabolic acidosis and potentially attenuated the prognostic association between markedly reduced SBE and outcomes. Secondly, SBE was assessed on only one occasion during the early phase of ICU admission, which limited the ability to capture its dynamic trajectory during the progression of SA‐AKI. Serial measurements of SBE and evaluation of its longitudinal changes may provide additional prognostic insights and should be explored in future studies. Thirdly, as a composite indicator influenced by various pathological processes, SBE is not specific to SA‐AKI and may also be reduced in conditions such as multiorgan dysfunction, lactic acidosis, or other acidotic states. This necessitates further investigation into its specificity. Additionally, although the MIMIC database offers high‐quality, structured data, it originates from a single U.S. institution, and differences in racial composition, healthcare resources, and treatment practices may limit the generalizability of the findings to other settings. It is evident that further multicenter, prospective studies are required in order to validate the applicability and robustness of SBE as a prognostic marker in a variety of clinical environments.

## 5. Conclusion

In summary, SBE, as a marker of metabolic acid–base balance, independently predicts both short‐ and long‐term mortality in patients with SA‐AKI, with a particularly strong prognostic value in subgroups such as younger and critically ill individuals. This study builds on extant evidence and supports the incorporation of SBE into clinical assessment tools to enhance disease severity evaluation and guide personalized treatment strategies. The incorporation of SBE thus offers valuable insights into the optimized management of critically ill patients.

**Nomenclature**
95% CIs: Confidence intervals
ADQI: Acute disease quality initiative
APSIII: Acute Physiology Score III
BE: Base excess
CCI: Charlson Comorbidity Index
CKD: Chronic kidney disease
DOD: Date of death
ESKD: End‐stage renal disease
HRs: Hazard ratios
ICU: Intensive care unit
KDIGO: Kidney disease: Improving global outcomes
MIMIC: Medical Information Mart for Intensive Care IV
RCS: Restricted cubic spline
RRT: Renal replacement therapy
SA‐AKI: Sepsis‐associated acute kidney injury
SBE: Standard base excess
SD: Standard deviation
SOFA: Sequential organ failure assessment
SQL: Structured query language
STROBE: Strengthening the Reporting of Observational Studies in Epidemiology

## Author Contributions

Mingming Li and Yuxia Li contributed equally to this work.

Mingming Li: conceptualization (lead), data curation (lead), formal analysis (lead), and writing–original draft (equal).

Yuxia Li: conceptualization (lead), data curation (lead), formal analysis (lead), and writing–original draft (equal).

Jingru Lan: data curation (supporting), methodology (equal), and validation (equal).

Mei Feng: data curation (supporting), methodology (equal), and validation (equal).

Jun Lyu: conceptualization (supporting), resources (lead), supervision (lead), and writing–review and editing (equal).

Xuling Li: conceptualization (supporting), methodology (supporting), validation (lead), supervision (equal), and writing–review and editing (lead).

All authors were actively involved in drafting the manuscript and critically revising it for important intellectual content. Jun Lyu and Xuling Li serve as corresponding authors, responsible for communication and ensuring the integrity of the work.

## Funding

This research did not receive any specific grant from funding agencies in the public, commercial, or not‐for‐profit sectors.

## Disclosure

All authors gave final approval of the version to be published and agreed to be accountable for all aspects of the work.

## Ethics Statement

The Medical Information Mart for Intensive Care IV (MIMIC‐IV) database was supported by grants from the National Institute of Biomedical Imaging and Bioengineering (NIBIB) of the National Institutes of Health (NIH) under award numbers R01‐EB001659 (2003–2013) and R01‐EB017205 (2014–2018) and approved by the Institutional Review Boards of Beth Israel Deaconess Medical Center (Boston, MA) and the Massachusetts Institute of Technology (Cambridge, MA). The present study is conducted in strict accordance with the Strengthening the Reporting of Observational Studies in Epidemiology guidelines and adheres to the ethical standards set forth in the Declaration of Helsinki.

## Consent

Data extracted from the MIMIC‐IV database do not require individual informed consent because the research data are publicly available, and all patient data are deidentified.

## Conflicts of Interest

The authors declare no conflicts of interest.

## Supporting Information

Additional supporting information can be found online in the Supporting Information section.

## Supporting information


**Supporting Information** Supporting Table 1: Definition/codes of the covariates. Supporting Table 2: Subgroup analysis results in secondary outcome. Supporting Figure 1: Inclusion and exclusion criteria for this study. Abbreviations: ICU: intensive care unit; MIMIC‐IV: Medical Information Mart for Intensive Care IV. Note: Participants were categorized into quartiles based on serum SBE levels as follows: Quartile 1 (SBE ≤ −5.42; range: −29.7 to −5.42), Quartile 2 (−5.42 < SBE ≤ −1.96), Quartile 3 (−1.96 < SBE ≤ 1.04), Quartile 4 (SBE > 1.04; range: 1.04–25.7). Supporting Figure 2: Proportion of missing data.

## Data Availability

The data were available on the MIMIC‐IV website at https://mimic.physionet.org/. The data in this article can be reasonably applied to the corresponding author.

## References

[1] Jt P. and Jl K. , Sepsis Associated Acute Kidney Injury, BMJ. (2019) 364, 10.1136/bmj.k4891. PMC6890472 30626586

[2] Zarbock A. , Nadim M. K. , Pickkers P. et al., Sepsis-Associated Acute Kidney Injury: Consensus Report of the 28th Acute Disease Quality Initiative Workgroup, Nature Reviews Nephrology. (2023) 19, no. 6, 401–417, 10.1038/s41581-023-00683-3. 36823168

[3] Peerapornratana S. , Manrique-Caballero C. L. , Gómez H. , and Kellum J. A. , Acute Kidney Injury from Sepsis: Current Concepts, Epidemiology, Pathophysiology, Prevention and Treatment, Kidney International. (2019) 96, no. 5, 1083–1099, 10.1016/j.kint.2019.05.026. PMC6920048 31443997

[4] White K. C. , Serpa-Neto A. , Hurford R. et al., Sepsis-Associated Acute Kidney Injury in the Intensive Care Unit: Incidence, Patient Characteristics, Timing, Trajectory, Treatment, and Associated Outcomes. A Multicenter, Observational Study, Intensive Care Medicine. (2023) 49, no. 9, 1079–1089, 10.1007/s00134-023-07138-0. PMC10499944 37432520

[5] Chua H. R. , Wong W. K. , Ong V. H. et al., Extended Mortality and Chronic Kidney Disease After Septic Acute Kidney Injury, Journal of Intensive Care Medicine. (2020) 35, no. 6, 527–535, 10.1177/0885066618764617. 29552953

[6] Zander R. , Base Excess (BE): Reloaded, European Journal of Medical Research. (2024) 29, no. 1, 10.1186/s40001-024-01796-6. PMC11089692 38735983

[7] Berend K. , Diagnostic Use of Base Excess in acid-base Disorders, New England Journal of Medicine. (2018) 378, no. 15, 1419–1428, 10.1056/NEJMra1711860. 29641969

[8] Smuszkiewicz P. , Jawień N. , Szrama J. , Lubarska M. , Kusza K. , and Guzik P. , Admission Lactate Concentration, Base Excess, and Alactic Base Excess Predict the 28-day Inward Mortality in Shock Patients, Journal of Clinical Medicine. (2022) 11, no. 20, 10.3390/jcm11206125. PMC9604570 36294445

[9] Luo C. , Duan Z. , Zheng T. et al., Base Excess is Associated with the Risk of all-cause Mortality in Critically Ill Patients with Acute Myocardial Infarction, Frontiers in Cardiovascular Medicine. (2022) 9, 10.3389/fcvm.2022.942485. PMC9396255 36017092

[10] Cheng Y. , Zhang Y. , Tu B. et al., Association Between Base Excess and Mortality Among Patients in ICU with Acute Kidney Injury, Frontiers of Medicine. (2021) 8, 10.3389/fmed.2021.779627. PMC8674681 34926523

[11] Wu W. T. , Li Y. J. , Feng A. Z. et al., Data Mining in Clinical Big Data: the Frequently Used Databases, Steps, and Methodological Models, Military Medical Research. (2021) 8, no. 1, 10.1186/s40779-021-00338-z. PMC8356424 34380547

[12] Johnson A. E. W. , Bulgarelli L. , Shen L. et al., MIMIC-IV, a Freely Accessible Electronic Health Record Dataset, Scientific Data. (2023) 10, no. 1, 10.1038/s41597-022-01899-x. PMC9810617 36596836

[13] von E. E. , Altman D. G. , Egger M. , Pocock S. J. , Gøtzsche P. C. , and Vandenbroucke J. P. , The Strengthening the Reporting of Observational Studies in Epidemiology (STROBE) Statement: Guidelines for Reporting Observational Studies, Lancet. (2007) 370, no. 9596, 1453–1457, 10.1016/S0140-6736(07)61602-X. 18064739

[14] Singer M. , Deutschman C. S. , Seymour C. W. et al., The Third International Consensus Definitions for Sepsis and Septic Shock (sepsis-3), JAMA. (2016) 315, no. 8, 801–810, 10.1001/jama.2016.0287. PMC4968574 26903338

[15] Khwaja A. , KDIGO Clinical Practice Guidelines for Acute Kidney Injury, Nephron Clinical Practice. (2012) 120, no. 4, c179–c184, 10.1159/000339789. 22890468

[16] Li X. , Tang Y. , Deng X. et al., Modified Frailty Index Effectively Predicts Adverse Outcomes in Sepsis Patients in the Intensive Care Unit, Intensive and Critical Care Nursing. (2024) 84, 10.1016/j.iccn.2024.103749. 38896964

[17] Park M. , Taniguchi L. U. , Noritomi D. T. , Libório A. B. , Maciel A. T. , and Cruz-Neto L. M. , Clinical Utility of Standard Base Excess in the Diagnosis and Interpretation of Metabolic Acidosis in Critically Ill Patients, Brazilian Journal of Medical and Biological Research. (2008) 41, no. 3, 241–249, 10.1590/S0100-879X2006005000199. 18097497

[18] Kraut J. A. and Madias N. E. , Metabolic Acidosis: Pathophysiology, Diagnosis and Management, Nature Reviews Nephrology. (2010) 6, no. 5, 274–285, 10.1038/nrneph.2010.33. 20308999

[19] Achanti A. and Szerlip H. M. , Acid-Base Disorders in the Critically Ill Patient, Clinical Journal of the American Society of Nephrology. (2023) 18, no. 1, 102–112, 10.2215/CJN.04500422. PMC10101555 35998977

[20] Hu J. , Wang Y. , Geng X. et al., Metabolic Acidosis as a Risk Factor for the Development of Acute Kidney Injury and Hospital Mortality, Experimental and Therapeutic Medicine. (2017) 13, no. 5, 2362–2374, 10.3892/etm.2017.4292. PMC5443206 28565850

[21] Do C. , Vasquez P. C. , and Soleimani M. , Metabolic Alkalosis Pathogenesis, Diagnosis, and Treatment: Core Curriculum 2022, American Journal of Kidney Diseases: The Official Journal of the National Kidney Foundation. (2022) 80, no. 4, 536–551, 10.1053/j.ajkd.2021.12.016. PMC10947768 35525634

[22] Curtis L. M. , Sex and Gender Differences in AKI, Kidney360. (2024) 5, no. 1, 160–167, 10.34067/KID.0000000000000321. PMC10833607 37990360

[23] Hamid A. K. , Pastor Arroyo E. M. , Calvet C. et al., Phosphate Restriction Prevents Metabolic Acidosis and Curbs Rise in FGF23 and Mortality in Murine Folic acid-induced AKI, Journal of the American Society of Nephrology. (2024) 35, no. 3, 261–280, 10.1681/ASN.0000000000000291. PMC10914210 38189228

[24] Yagi K. and Fujii T. , Management of Acute Metabolic Acidosis in the ICU: Sodium Bicarbonate and Renal Replacement Therapy, Critical Care. (2021) 25, no. 1, 10.1186/s13054-021-03677-4. PMC8406840 34461963

[25] Levraut J. and Grimaud D. , Treatment of Metabolic Acidosis, Current Opinion in Critical Care. (2003) 9, no. 4, 260–265, 10.1097/00075198-200308000-00002. 12883279

[26] Schork A. , Moll K. , Haap M. , Riessen R. , and Wagner R. , Course of Lactate, Ph and Base Excess for Prediction of Mortality in Medical Intensive Care Patients, PLoS One. (2021) 16, no. 12, 10.1371/journal.pone.0261564. PMC8687550 34929006

[27] Heldeweg M. L. A. , Langer T. , and Duška F. , Guiding Resuscitation in Shock: Base Excess or Lactate?, Critical Care. (2024) 28, no. 1, 10.1186/s13054-024-05039-2. PMC11264676 39026216

[28] Li Z. , Qian D. , Xia Y. , Kang K. , and Feng T. , Associations Between Base Excess, Alactic Base Excess, and Kidney Function Deterioration in Patients Undergoing Coronary Artery Bypass Grafting Surgery: a Retrospective Cohort Study, Journal of Cardiothoracic and Vascular Anesthesia. (2025) 39, no. 25, S1053–S1770, 10.1053/j.jvca.2025.03.033. 40221311

[29] Yuan J. , Liu X. , Liu Y. et al., Association Between Base Excess and 28-day Mortality in Sepsis Patients: a Secondary Analysis Based on the MIMIC- IV Database, Heliyon. (2023) 9, no. 5, 10.1016/j.heliyon.2023.e15990. PMC10199177 37215834

[30] Zante B. , Reichenspurner H. , Kubik M. , Kluge S. , Schefold J. C. , and Pfortmueller C. A. , Base Excess is Superior to lactate-levels in Prediction of ICU Mortality After Cardiac Surgery, PLoS One. (2018) 13, no. 10, 10.1371/journal.pone.0205309. PMC6173442 30289956

