# Supplementary material for: Prognostic Value of Standard Base Excess in Sepsis‐Associated Acute Kidney Injury: A Retrospective Cohort Study
Source: Int J Nephrol. 2026 Sep 22;2026:8851195. doi: 10.1155/ijne/8851195 (PMC13596010; doi:10.1155/ijne/8851195)
Supplement: Supplementary file 1 [file IJNE-2026-8851195-s001.zip › 8851195.f1.docx]

**Supplementary Table 1** Definition/codes of the covariates

| **Variables** | **Codes/definition** |
| --- | --- |
| Age | Continuous variable |
| Sex | Binary variable (1 = Male; 0 = Female) |
| Race | Binary variable (1 = White; 0 = Other&) |
| SOFA | Continuous variable |
| APSIII | Continuous variable |
| CCI | Continuous variable |
| Hypertension | Binary variable (1 = Yes; 0 = No) |
| Diabetes | Binary variable (1 = Yes; 0 = No) |
| Septic shock | Binary variable (1 = Yes; 0 = No) |
| Chronic kidney disease | Binary variable (1 = Yes; 0 = No) |
| Vasoactive agent | Binary variable (1 = Yes; 0 = No) |
| Mechanical ventilation | Binary variable (1 = Yes; 0 = No) |
| RRT | Binary variable (1 = Yes; 0 = No) |

Abbreviations: SOFA, Sequential Organ Failure Assessment; APSIII, Acute Physiology Score III; CCI, Charlson Comorbidity Index; RRT, renal replacement therapy.

**Supplementary Table 2** Subgroup analysis results in secondary outcome

| **Subgroups** | **SBE** | **HR (95%CI)** | **P-value** | **P for interaction** |
| --- | --- | --- | --- | --- |
| **Age** | | | | |
| <67 | Quartile 1 | Reference | | |
|  | Quartile 2 | 0.80 (0.70-0.90) | <0.001* | 0.259 |
|  | Quartile 3 | 0.77 (0.68-0.88) | <0.001* | 0.227 |
|  | Quartile 4 | 0.84 (0.73-0.95) | 0.007* | 0.011 |
| >=67 | Quartile 1 | Reference | | |
|  | Quartile 2 | 0.85 (0.77-0.93) | <0.001* | 0.259 |
|  | Quartile 3 | 0.83 (0.76-0.92) | <0.001* | 0.227 |
|  | Quartile 4 | 0.99 (0.90-1.08) | 0.744 | 0.011 |
| **Sex** | | | | |
| Female | Quartile 1 | Reference | | |
|  | Quartile 2 | 0.79 (0.71-0.89) | <0.001* | 0.913 |
|  | Quartile 3 | 0.83 (0.74-0.94) | 0.002* | 0.092 |
|  | Quartile 4 | 0.95 (0.86-1.06) | 0.392 | 0.049 |
| Male | Quartile 1 | Reference | |  |
|  | Quartile 2 | 0.82 (0.75-0.91) | <0.001* | 0.913 |
|  | Quartile 3 | 0.77 (0.70-0.85) | <0.001* | 0.092 |
|  | Quartile 4 | 0.88 (0.79-0.97) | 0.010* | 0.049 |
| **Race** | | | | |
| White | Quartile 1 | Reference | | |
|  | Quartile 2 | 0.78 (0.71-0.86) | <0.001* | 0.052 |
|  | Quartile 3 | 0.82 (0.75-0.91) | <0.001* | 0.480 |
|  | Quartile 4 | 0.92 (0.84-1.01) | 0.095 | 0.967 |
| Others | Quartile 1 | Reference | | |
|  | Quartile 2 | 0.87 (0.77-0.98) | 0.020* | 0.052 |
|  | Quartile 3 | 0.74 (0.65-0.84) | <0.001* | 0.480 |
|  | Quartile 4 | 0.88 (0.78-1.00) | 0.051 | 0.967 |
| SOFA | | | | |
| <10 | Quartile 1 | Reference | | |
|  | Quartile 2 | 0.88 (0.80-0.96) | 0.005* | 0.035 |
|  | Quartile 3 | 0.85 (0.77-0.94) | 0.001* | 0.069 |
|  | Quartile 4 | 0.99 (0.90-1.08) | 0.757 | 0.013 |
| >=10 | Quartile 1 | Reference | | |
|  | Quartile 2 | 0.75 (0.66-0.84) | <0.001* | 0.035 |
|  | Quartile 3 | 0.72 (0.63-0.83) | <0.001* | 0.069 |
|  | Quartile 4 | 0.78 (0.68-0.90) | 0.001* | 0.013 |

Abbreviations: SBE: standard base excess; HR: hazard ratio; CI: confidence intervals; SOFA: sequential organ failure assessment.

Note:

Participants were categorized into quartiles based on serum SBE levels as follows:

Quartile 1 (SBE ≤ -5.42; range: -29.7 to -5.42),

Quartile 2 (-5.42 < SBE ≤ -1.96),

Quartile 3 (-1.96 < SBE ≤ 1.04),

Quartile 4 (SBE > 1.04; range: 1.04 to 25.7).

*Significant difference between patients in four groups (p < 0.05).

**Supplementary Figure 1** Inclusion and exclusion criteria for this study

Abbreviations: ICU: intensive care unit; MIMIC-IV: Medical Information Mart for Intensive Care IV.

Note: Participants were categorized into quartiles based on serum SBE levels as follows:

Quartile 1 (SBE ≤ -5.42; range: -29.7 to -5.42),

Quartile 2 (-5.42 < SBE ≤ -1.96),

Quartile 3 (-1.96 < SBE ≤ 1.04),

Quartile 4 (SBE > 1.04; range: 1.04 to 25.7).

**Supplementary Figure 2** Proportion of missing data
